# The Downlink Performance for Cell-Free Massive MIMO with Instantaneous CSI in Slowly Time-Varying Channels

**DOI:** 10.3390/e23111552

**Published:** 2021-11-22

**Authors:** Tongzhou Han, Danfeng Zhao

**Affiliations:** College of Information and Communication Engineering, Harbin Engineering University, Harbin 150001, China; zhaodanfeng@hrbeu.edu.cn

**Keywords:** cell-free massive MIMO, FDD, conjugate beamforming, downlink transmission, power control

## Abstract

In centralized massive multiple-input multiple-output (MIMO) systems, the channel hardening phenomenon can occur, in which the channel behaves as almost fully deterministic as the number of antennas increases. Nevertheless, in a cell-free massive MIMO system, the channel is less deterministic. In this paper, we propose using instantaneous channel state information (CSI) instead of statistical CSI to obtain the power control coefficient in cell-free massive MIMO. Access points (APs) and user equipment (UE) have sufficient time to obtain instantaneous CSI in a slowly time-varying channel environment. We derive the achievable downlink rate under instantaneous CSI for frequency division duplex (FDD) cell-free massive MIMO systems and apply the results to the power control coefficients. For FDD systems, quantized channel coefficients are proposed to reduce feedback overhead. The simulation results show that the spectral efficiency performance when using instantaneous CSI is approximately three times higher than that achieved using statistical CSI.

## 1. Introduction

The global annual wireless data traffic continues to grow rapidly. Devices are growing at a compound annual growth rate (CAGR) of approximately 10%, approaching 30 billion devices by 2023 [1]. To support the massive data traffic and connectivity in the future, new technologies will be discussed and compared in the development of sixth generation (6G) standards to surpass existing wireless communication systems. Since cell-free massive multiple-input multiple-output (MIMO) was proposed, it has been widely investigated by the academic community, and it is considered a key technology of future 6G [2,3]. Cell-free massive MIMO systems, in which users are simultaneously served by many access points (APs), can provide uniform quality of service (QoS) to users in a wide area, ensuring the same experience for all users [4]. All APs are connected to a central processing unit (CPU) and cooperate to cover a designated area. Because the system has no cell boundaries, cell-free massive MIMO can overcome the handover problem in traditional cellular communication systems. In addition, cell-free massive MIMO has the advantages of high coverage probability, substantial spectral efficiency, and high energy efficiency [5,6].

Cell-free massive MIMO, allowing a user-centric approach, deploys many APs to serve a small number of users simultaneously in a specific area. The distance between users and APs is shortened to reduce path loss, which reduces the impact of large-scale fading. The flexible deployment of low-cost APs allows flexible deployment in space-constrained scenarios, such as roadside facilities and building walls. The cell-free architecture avoids handover in cellular networks and reduces delays and network outages caused by handover [7]. The spectral efficiency of cell-free massive MIMO systems with conjugate beamforming (CB) and matched filter (MF) receivers shows a 5–10 times improvement in throughput per user compared to small cells and is not affected by correlated shadow fading [8]. The zero forcing (ZF) precoder reduces interuser interference and further improves the average user throughput, and the energy efficiency of the ZF precoder is shown to be significantly improved over conventional centralized massive MIMO systems [9]. Moreover, cell-free achieves high energy efficiency under different path loss models [10].

Benefiting from the channel hardening phenomenon, massive MIMO uses linear processing to achieve excellent performance and eliminate small-scale fading effects [11]. In time division duplex (TDD)-based centralized massive MIMO systems, due to the channel hardening effect, downlink channel estimation is not required, and the channel state information (CSI) of uplink channel estimation is directly precoded in downlink transmission [12]. Relying on the channel hardening properties inherited from massive MIMO, all cell-free massive MIMO systems adopt simple precoding and combining schemes [8,13]. Performance analysis approaches related to centralized massive MIMO are widely applied. Many works use statistical CSI for cell-free massive MIMO power control [8,13,14,15]. The advantage of this approach is that large-scale fading tends to change slowly, so statistical information can reduce the amount of calculation. When the channel hardening phenomenon is evident, power control within statistical CSI is almost identical to instant information. According to the literature [16], cell-free massive MIMO with single-antenna APs experiences a weaker channel hardening effect. In this case, if power control uses the statistical channel information, then the result will be far from optimal.

Unlike TDD cell-free MIMO, the frequencies of uplink and downlink channels are different in frequency division duplex (FDD) systems. There is no reciprocity between uplink and downlink channels [17,18]. Downlink training is required to obtain downlink CSI. In the slow-changing channel case, the CSI feedback will be much less than that of the fast-changing channel, and the channel acquisition overhead is acceptable. If cell-free massive MIMO is deployed on a campus, in a conference venue or in other walking-oriented scenarios, where users move slowly and the channel coherence time is long [19], then the feedback of downlink CSI is lower, and there is sufficient time for estimation and feedback. This paper proposes that in a slowly time-varying channel environment, both the CPU and user equipment (UE) have sufficient time to obtain instantaneous CSI to use for power control. When the system is deployed in a location where people are walking or sitting, such as on a university campus, for large gatherings, or in large conference venues, the slowly time-varying fading channel condition can be realized when the UE moves slowly [20].

The main results of this work are as follows. We elaborate on the channel hardening and favorable propagation of the cell-free massive MIMO channel and the centralized massive MIMO channel [11]. We note that the favorable conditions in the cell-free massive MIMO channel still exist. The channels between users are orthogonal; however, the channel hardening phenomenon is not as evident as that in centralized massive MIMO [16]. Based on this fact, we propose to use accurate instantaneous CSI instead of statistical CSI for downlink transmission and power control. First, we use the instantaneous CSI to analyze the achievable rate of downlink transmission considering the channel estimation and apply the result to power control. Second, to reduce the feedback overhead, we quantize the estimated CSI with low precision and consider the effect of quantization error in the power control. Finally, we compare the performance of FDD cell-free massive MIMO with instantaneous CSI to that of other schemes under the urban microcell (UMi) scenario.

The rest of this paper is organized as follows. In Section 2, we describe the FDD cell-free massive MIMO system model. In Section 3, we present the CB and power control with instantaneous CSI for downlink transmission. The quantization for feedback and power control with different quantized bits is developed in Section 4. We provide numerical results and discussions in Section 5 and finally conclude the paper in Section 6.

*Notations*: Boldface letters G and g denote matrix and column vectors. The superscripts ${\left(\right)}^{*}$, ${\left(\right)}^{*}T$, and ${\left(\right)}^{H}$ stand for the conjugate, transpose, and conjugate-transpose, respectively. ∥.∥ and E{.} denote the Euclidean norm and the expectation operators, respectively. $a∼\mathrm{CN}(0,\sigma^{2})$ denotes a circularly symmetric complex Gaussian random variable *a* with zero mean and variance $\sigma^{2}$. ℜ(.) and ℑ(.) are the real and imaginary parts of a complex matrix, respectively. ∘ stands for the Hadamard product, and vec(.) indicates vectorization of a matrix.

## 2. System Model

**Figure 1:**
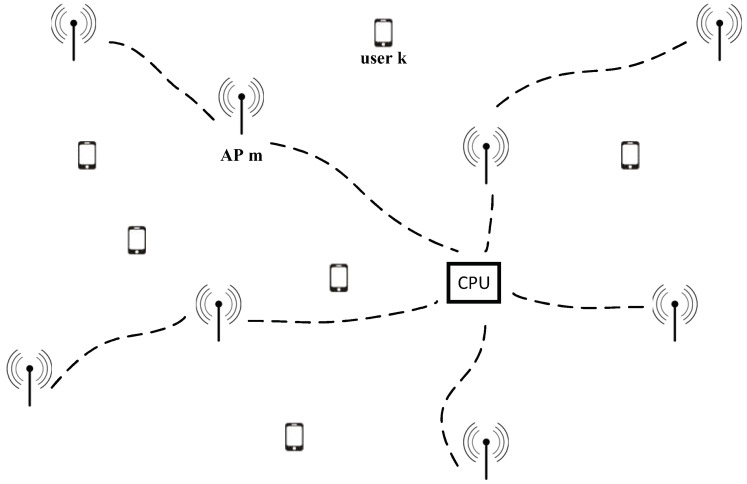
In a cell-free massive MIMO, APs and UEs are equipped with one antenna.

In a cell-free massive MIMO system in a finite-size network region, there are *M* APs with a single antenna distributed randomly in the region, and the APs serve *K* terminals equipped with a single antenna, M≫K. We assume that all APs are connected to a CPU via an unspecified backhaul network, as shown in Figure 1. The wireless channel changes slowly, and the channel model is the fading channel model in which fading coefficients are constant over time-frequency blocks. For the sake of simplicity, we omit the indexes of different time-frequency blocks. The channel gain $g_{mk}=\sqrt{\beta_{mk}}h_{mk}$ between the *m*th AP and the *k*th terminal remains constant in a coherence time block $\tau_{c}$ and a coherence bandwidth block $B_{c}$, where $\beta_{mk}$ and $h_{mk}$ are the large-scale and small-scale fading coefficients, respectively. The channel vector of the *k*th terminal is

(1) $$g_{k}={\left[g_{1k},g_{2k},\cdots ,g_{Mk}\right]}^{T}={\left[\sqrt{\beta_{1k}}h_{1k},\sqrt{\beta_{2k}}h_{2k},\cdots ,\sqrt{\beta_{Mk}}h_{Mk}\right]}^{T},$$

and

(2) $$G=\left[g_{1},g_{2},\cdots ,g_{K}\right].$$

The large-scale fading $\beta_{mk}$ is affected by path loss and shadow fading between the AP and terminal and the small-scale fading coefficients $h_{mk}$ are independent and identically distributed (i.i.d.) and satisfy $h_{mk}∼\mathrm{CN}\left(0,1\right)$. Furthermore, the large-scale fading coefficients are assumed to be known as needed.

**Figure 2:**
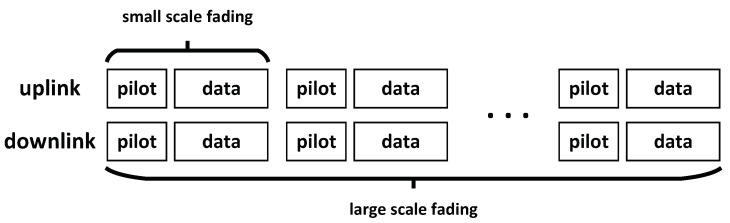
In FDD system, uplink and downlink channels are different, and CSIs are estimated separately.

We assume that the terminal does not move quickly in the considered scenario. When the correlation time is greater than the channel feedback delay, which is typically sub-millisecond, the channel is considered to be slow-varying. In other words, we assume that the terminal has sufficient time to feedback the CSI to the AP in FDD system. Consider the frequency $f_{c}=2.0$ GHz, the system bandwidth B=10 MHz, and the orthogonal frequency division multiplexing (OFDM) size N=2048. The maximum delay spread is $\tau_{max}=5\;\mu$s. The velocity of users is v=2 m/s. The coherence time is 31.7 ms. There are 6184 samples in a coherence time-frequency block [19,21]. Section 2.1 notes that the channel is less hardened in cell-free massive MIMO systems. Therefore, both the transmitter and the receiver obtain the instantaneous CSI, as shown in Figure 2. Although the overhead of feedback of the CSI increases, the simulation results show that the spectral efficiency is substantially increased.

### 2.1. Channel Hardening

Channel hardening phenomenon is the phenomenon where the variance of the mutual information of the MIMO channel changes slowly as the number of antennas grows [22,23]. Channel hardening can bring several advantages in large dimensional signal processing. As the number of antennas increases, the channel hardening phenomenon makes the multi-antenna fading channel almost deterministic. In centralized massive MIMO systems, this phenomenon is exploited to obtain high spectral efficiency and high reliability. Cell-free massive MIMO distributes AP antennas randomly in a scene, and the large-scale fading between each AP and the terminal is no longer the same. With one antenna per AP, increasing the AP density does not lead to channel hardening. Equation (3) is the channel gain variation to prove channel hardening [16,24].

(3) $$\mathrm{Var}\left[\frac{∥g_{k}{∥}^{2}}{E[∥g_{k}{∥}^{2}|r]}\right]=\frac{\mathrm{Var}[∥g_{k}{∥}^{2}|r]}{(E[∥g_{k}{∥}^{2}{\left|r]\right)}^{2}}\to 0\;\mathrm{as}\;M\to \infty ,$$

where $g_{k}$ is the channel vector for the *k*th terminal, r is the distance vector between APs and the terminal, and *M* is the number of antennas. The channel gain is

(4) $${∥g_{k}∥}^{2}=g_{k}^{H}g_{k}=\sum_{m=1}^{M}\beta_{mk}h_{mk}^{*}h_{mk}.$$

When the positions of the antenna and the terminal are determined, $\mathrm{Var}\left[{∥g_{k}∥}^{2}|r\right]$ and $E\left[{∥g_{k}∥}^{2}|r\right]$ are as follows.

(5) $$\mathrm{Var}\left[{∥g_{k}∥}^{2}|r\right]=\sum_{m=1}^{M}\beta_{mk}^{2}\mathrm{Var}\left[h_{mk}^{*}h_{mk}\right],$$

and

(6) $$E\left[{∥g_{k}∥}^{2}|r\right]=\sum_{m=1}^{M}\beta_{mk}E\left[h_{mk}^{*}h_{mk}\right].$$

To analyze the difference in channel hardening between centralized massive MIMO and cell-free massive MIMO, the received power of the terminal is assumed to be normalized, $\sum_{m=1}^{M}\beta_{mk}=1$. Substitution of (5) and (6) into (3) yields

(7) $$\mathrm{Var}\left[\frac{{∥g_{k}∥}^{2}}{E\left[{∥g_{k}∥}^{2}|r\right]}\right]=\frac{\sum_{m=1}^{M}\beta_{mk}^{2}}{{\left(\sum_{m=1}^{M}\beta_{mk}\right)}^{2}}=\left\{\begin{array}{cc} \frac{1}{M}, & for\;\mathrm{centralized}\\ \sum_{m=1}^{M}\beta_{mk}^{2}, & for\;\mathrm{cell-free} \end{array}\right..$$

When $\beta_{1k}=\cdots =\beta_{Mk}$, $\sum_{m=1}^{M}\beta_{mk}^{2}$ is minimum. In cell-free massive MIMO systems, the large-scale fading will not be identical. Therefore, the channel hardening is not as good as that of the centralized massive MIMO system.

The variation in the channel gain of centralized massive MIMO and cell-free massive MIMO with the same number of antennas is shown in Table 1. As the number of antennas increases, the channel of the centralized massive MIMO system tends to be deterministic. However, this finding does not hold for the cell-free massive MIMO channel with the same number of antennas, where the channel gain is far less stable than that of the centralized massive MIMO system.

### 2.2. Favorable Propagation

Favorable propagation conditions are defined as mutual orthogonality between the channel vectors of different terminals, which is a key feature of massive MIMO. The asymptotically favorable propagation condition can be defined as follows [16]:

(8) $$\frac{g_{k}^{H}g_{j}}{\sqrt{E[∥g_{k}{∥}^{2}|r]E[∥g_{j}{∥}^{2}\left|r\right]}}\to 0,\mathrm{as}\;M\to \infty ,k\neq j.$$

Table 2 shows that, unlike the channel hardening phenomenon, the favorable propagation conditions of the cell-free massive MIMO system have not diminished. In regard to centralized massive MIMO, the channel vectors between terminals become increasingly orthogonal as the number of antennas increases.

### 2.3. Downlink Transmission and Minimum Mean Square Error (MMSE) Estimation

**Figure 3:**
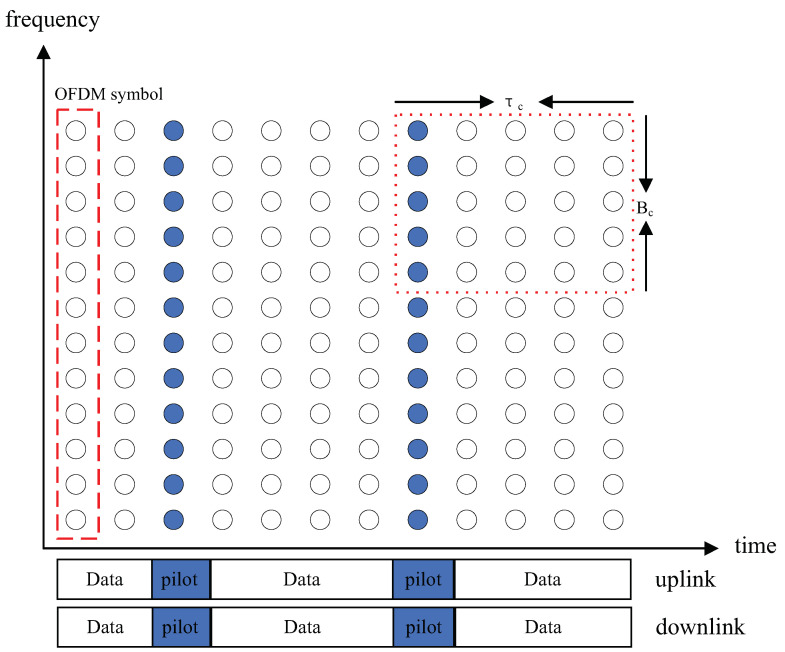
The pilot is in a block within a $\tau_{c}$ and $B_{c}$, and in different blocks, pilot will be multiplexing.

For the *n*th subchannel that contains several subcarriers and the received signal, $y_{k,n}$ of the *k*th terminal can be expressed as

(9) $$y_{k,n}=g_{k,n}^{T}x_{n}+w_{k,n},$$

where $g_{k,n}\in C^{M\times 1}$, and $x_{n}\in C^{M\times 1}$ is the transmitted precoded signal or pilot signal. For convenience, we only consider the channel estimation on a subchannel. For example, a block as shown in Figure 3.

In the CSI acquisition phase, the *i*th AP sends pilot sequences $\varphi_{i}\in C^{\tau_{p}}$, and $\varphi_{i}^{H}\varphi_{j}=\delta_{ij}$. In FDD cell-free massive MIMO, downlink CSI is estimated at terminals and is fed back to the CPU. Pilots $\varphi_{1},\cdots ,\varphi_{M}$ are assigned for each AP, $\tau_{p}=M$, and the pilot transmit power limit is $\rho =\rho_{ap}$. The MMSE estimate of $g_{mk}$ is

(10) $${\hat{g}}_{mk}=c_{mk}\varphi_{m}^{H}\left(\sqrt{\rho \tau_{p}}\sum_{j=1}^{M}g_{jk}\varphi_{j}+w_{k}\right),$$

where $c_{mk}=\frac{\sqrt{\rho \tau_{p}}\beta_{mk}}{1+\rho \tau_{p}\beta_{mk}}$. $w_{k}∼\mathrm{CN}\left(0,I\sigma_{w}\right)$. ${\tilde{g}}_{mk}=g_{mk}-{\hat{g}}_{mk}$ is the channel estimation error. ${\hat{g}}_{mk}$ and ${\tilde{g}}_{mk}$ are uncorrelated, with

(11) $${\hat{g}}_{mk}∼\mathrm{CN}\left(0,\gamma_{mk}\right)$$

and

(12) $${\tilde{g}}_{mk}∼\mathrm{CN}\left(0,\beta_{mk}-\gamma_{mk}\right),$$

where $\gamma_{mk}=\frac{\rho \tau_{p}\beta_{mk}^{2}}{\sigma_{w}^{2}+\rho \tau_{p}\beta_{mk}}$.

The estimated CSI is fed back to the APs via the uplink and is used during precoding and power control. It is assumed that there is sufficient time for downlink channel estimation and CSI feedback in a slowly varying channel environment.

## 3. CB and Power Control

The channel hardening phenomenon tends to determine the gain of the random channel vector. Cell-free massive MIMO with a single-antenna AP diminishes the channel hardening phenomenon. Therefore, in this section, we propose to use instantaneous CSI for precoding and power control instead of the statistical information. In a slowly varying channel environment, there are sufficient resources to feed the estimated downlink CSI to the AP and CPU.

### 3.1. CB with Instantaneous CSI

With CB precoding, the *m*th AP transmits the signal

(13) $$x_{m}=\sqrt{\rho_{ap}}\sum_{i=1}^{K}\sqrt{\eta_{mi}}{\hat{g}}_{mi}^{*}s_{i},$$

where $\rho_{ap}$ is the transmit power of each AP, and $s_{i}$ is the data signal to the *i*th terminal, with $E(|s_{i}{|}^{2})=1$. $\eta_{mi}$ is the power control coefficient. The signal received by the *k*th terminal is

(14) $$y_{k}=\sum_{m=1}^{M}g_{mk}x_{m}+w_{k}=DS_{k}+UI_{kk^{\prime }}+ES+w_{k},$$

where $DS_{k}$, $UI_{kk^{\prime }}$, and ES represent the strength of the desired signal, the interference caused by the $k^{\prime }$ terminal, and the error signal caused by the channel estimation error, respectively. $DS_{k}$, $UI_{kk^{\prime }}$, and ES are expressed as follows:

(15) $$DS_{k}=\sqrt{\rho_{ap}}\sum_{m=1}^{M}\sqrt{\eta_{mk}}{\hat{g}}_{mk}{\hat{g}}_{mk}^{*}s_{k},$$

(16) $$UI_{kk^{\prime }}=\sqrt{\rho_{ap}}\sum_{i\neq k}^{K}\sum_{m=1}^{M}\sqrt{\eta_{mi}}{\hat{g}}_{mk}{\hat{g}}_{mi}^{*}s_{i},$$

(17) $$ES=\sqrt{\rho_{ap}}\sum_{i=1}^{K}\sum_{m=1}^{M}\sqrt{\eta_{mi}}{\tilde{g}}_{mi}{\hat{g}}_{mi}^{*}s_{i}.$$

The gain, $\rho_{ap}{\left(\sum_{m=1}^{M}\sqrt{\eta_{mk}}{\hat{g}}_{mk}{\hat{g}}_{mk}^{*}\right)}^{2}$, obtained by the terminal via downlink is used for reception. Unlike the scheme in the previous literature [8,13,14,15], the channel hardening is no longer exploited, and channel statistical information, $\sqrt{\rho_{ap}}E\left[\sum_{m=1}^{M}\sqrt{\eta_{mk}}{\hat{g}}_{mk}{\hat{g}}_{mk}^{*}\right]$, is not used for reception. However, accurate estimated CSI participates in receiving processing. An achievable rate of the *k*th terminal with CB is $SE_{k}=log_{2}\left(1+{\mathrm{SIN}R}_{k}\right)$, and the expression of ${\mathrm{SIN}R}_{k}$ is shown as follows:

(18) $${\mathrm{SIN}R}_{k}=\frac{\rho_{ap}{\left(\sum_{m=1}^{M}\sqrt{\eta_{mk}}{\hat{g}}_{mk}{\hat{g}}_{mk}^{*}\right)}^{2}}{\rho_{ap}\sum_{i\neq k}^{K}{\left(\sum_{m=1}^{M}\sqrt{\eta_{mi}}{\hat{g}}_{mk}{\hat{g}}_{mi}^{*}\right)}^{2}+\rho_{ap}\sum_{i=1}^{K}\sum_{m=1}^{M}\eta_{mi}{\hat{g}}_{mi}{\hat{g}}_{mi}^{*}{\tilde{g}}_{mk}{\tilde{g}}_{mk}^{*}+\sigma_{w}^{2}}.$$

${\tilde{g}}_{mk}$ is the channel estimation error and cannot be obtained accurately. Therefore, substitute $E[{\tilde{g}}_{mk}{\tilde{g}}_{mk}^{*}]$ for ${\tilde{g}}_{mk}{\tilde{g}}_{mk}^{*}$ as follows:

(19) $${\mathrm{SIN}R}_{k}=\frac{\rho_{ap}{\left(\sum_{m=1}^{M}\sqrt{\eta_{mk}}{\hat{g}}_{mk}{\hat{g}}_{mk}^{*}\right)}^{2}}{\rho_{ap}\sum_{i\neq k}^{K}{\left(\sum_{m=1}^{M}\sqrt{\eta_{mi}}{\hat{g}}_{mk}{\hat{g}}_{mi}^{*}\right)}^{2}+\rho_{ap}\sum_{i=1}^{K}\sum_{m=1}^{M}\eta_{mi}{\hat{g}}_{mi}{\hat{g}}_{mi}^{*}E\left[{\tilde{g}}_{mk}{\tilde{g}}_{mk}^{*}\right]+\sigma_{w}^{2}}.$$

The interference term in (19), ${\left(\sum_{m=1}^{M}\sqrt{\eta_{mi}}{\hat{g}}_{mk}{\hat{g}}_{mi}^{*}\right)}^{2}$, appears complex in power control calculations, and optimization tools, such as convex programming (CVX), cannot process such terms directly. Thus, the real part and the imaginary part are handled separately.

(20) $${\left(\sum_{m=1}^{M}\sqrt{\eta_{mi}}{\hat{g}}_{mk}{\hat{g}}_{mi}^{*}\right)}^{2}={\left(\sum_{m=1}^{M}\sqrt{\eta_{mi}}ℜ\left({\hat{g}}_{mk}{\hat{g}}_{mi}^{*}\right)\right)}^{2}+{\left(\sum_{m=1}^{M}\sqrt{\eta_{mi}}ℑ\left({\hat{g}}_{mk}{\hat{g}}_{mi}^{*}\right)\right)}^{2}.$$

### 3.2. Power Control

With max–min power control, cell-free massive MIMO provides the same QoS to the terminals in the service area. We propose that instantaneous CSI participate in power control in a slowly varying channel environment to avoid performance loss due to insufficient channel hardening.

The max–min power allocation problem for downlink transmission is as follows:

(21) $$\begin{array}{cc} \max_{\eta } & \min_{k}\;SE_{k}\\ s.t. & \;\sum_{i=1}^{K}\eta_{mi}{\hat{g}}_{mi}{\hat{g}}_{mi}^{*}\leq 1,m=1,\cdots ,M\\ \; & \eta_{mi}\geq 0,m=1,\cdots ,M,i=1,\cdots ,K. \end{array}$$

$SE_{k}$ is replaced with (19) and (20). This problem is solved via the bisection method [25]. Set *t* as the target available rate for each terminal. Repeatedly use the bisection method to solve the following feasibility problem that obtains the power control coefficients.

(22) $$\begin{array}{cc} \mathrm{find} & \;\zeta \\ s.t. & \;\sqrt{\rho_{ap}}\left(\sum_{m=1}^{M}\zeta_{mk}{\hat{g}}_{mk}{\hat{g}}_{mk}^{*}\right)\geq \sqrt{t}∥[\alpha_{k}^{\left(1\right)},\alpha_{k}^{\left(2\right)},\alpha_{k}^{\left(3\right)},\sigma_{w}]∥,k=1,\cdots ,K\\ \; & \sum_{i=1}^{K}\zeta_{mi}^{2}{\hat{g}}_{mi}{\hat{g}}_{mi}^{*}\leq 1,m=1,\cdots ,M\\ \; & \zeta_{mi}\geq 0,m=1,\cdots ,M,i=1,\cdots ,K, \end{array}$$

where $\zeta_{mk}=\sqrt{\eta_{mk}}$, $\zeta =[{\left[\sqrt{\eta_{1,1}},\cdots ,\sqrt{\eta_{M,1}}\right]}^{T},\cdots ,{\left[\sqrt{\eta_{1,K}},\cdots ,\sqrt{\eta_{M,K}}\right]}^{T}]$, and $\alpha_{k}^{\left(1\right)}$, $\alpha_{k}^{\left(2\right)}$, and $\alpha_{k}^{\left(3\right)}$ are

(23) $$\alpha_{k}^{\left(1\right)}=ℜ\left(g_{k}^{T}\left(\zeta_{-k}\circ {\hat{G}}_{-k}\right)\right),$$

(24) $$\alpha_{k}^{\left(2\right)}=ℑ\left(g_{k}^{T}\left(\zeta_{-k}\circ {\hat{G}}_{-k}\right)\right),$$

(25) $$\alpha_{k}^{\left(3\right)}=vec\left(\zeta \circ abs\left(\hat{G}\right)\right)\sqrt{diag(\beta_{k}-\gamma_{k})}.$$

## 4. Quantization of CSI Feedback

In FDD, the terminal estimates the downlink CSI and feeds it back to the APs and CPU. To reduce the overhead of feedback, the estimated channel information is quantized before feedback.

(26) $${\hat{g}}_{mk}^{q}=Q\left({\hat{g}}_{mk}\right).$$

According to the Bussgang theorem, the quantized output is decomposed into a desired signal component and an uncorrelated distortion [26] as follows:

(27) $$\begin{array}{cc} {\hat{g}}_{mk}^{q} & =F_{mk}{\hat{g}}_{mk}+e_{mk}\\ \; & =F_{mk}\left(g_{mk}-{\tilde{g}}_{mk}\right)+e_{mk}\\ \; & =F_{mk}g_{mk}+e_{mk}^{\prime }, \end{array}$$

where $F_{mk}$ is obtained from the linear MMSE estimation, $F_{mk}=\left(1-\varsigma_{mk}\right)$, and $\varsigma_{mk}=\frac{E[{\left({\hat{g}}_{mk}-{\hat{g}}_{mk}^{q}\right)}^{2}]}{E[{\hat{g}}_{mk}^{2}]}$ is the distortion factor. The power of the non-Gaussian effective noise $e_{mk}^{\prime }$ is as follows:

(28) $$E\left[e_{mk}^{\prime }{\left(e_{mk}^{\prime }\right)}^{*}\right]=\left(1-\gamma_{mk}\right)\gamma_{mk}E\left[{\hat{g}}_{mk}^{2}\right]+{\left(1-\gamma_{mk}\right)}^{2}E\left[{\tilde{g}}_{mk}^{2}\right].$$

The number of quantization bits, *b*, is at least 1. As *b* increases, the quantization error ς decreases, and there is less loss of channel information due to quantization. Under the high-resolution assumption, i.e., more than 4 bits, approximately the following distortion exists:

(29) $$\varsigma \approx \frac{\pi \sqrt{3}}{2}2^{-2b}.$$

With CB precoding, the *m*th AP transmits the signal

(30) $$x_{m}^{q}=\sqrt{\rho_{ap}}\sum_{i=1}^{K}\sqrt{\eta_{mi}}{\left({\hat{g}}_{mi}^{q}\right)}^{*}s_{i},$$

and the signal received by the *k*th terminal is

(31) $$y_{k}=\sum_{m=1}^{M}g_{mk}x_{m}^{q}+w_{k}=DS_{k}^{q}+UI_{kk^{\prime }}^{q}+ES^{q},$$

where

(32) $$DS_{k}^{q}=\sqrt{\rho_{ap}}\sum_{m=1}^{M}\sqrt{\eta_{mk}}{\hat{g}}_{mk}^{q}{\left({\hat{g}}_{mk}^{q}\right)}^{*}s_{k},$$

(33) $$UI_{kk^{\prime }}^{q}=\sqrt{\rho_{ap}}\sum_{i\neq k}^{K}\sum_{m=1}^{M}\sqrt{\eta_{mi}}{\hat{g}}_{mk}^{q}\left({\hat{g}}_{mi}^{q}\right){}^{*}s_{i},$$

(34) $$ES^{q}=\sqrt{\rho_{ap}}\sum_{i=1}^{K}\sum_{m=1}^{M}\sqrt{\eta_{mi}}e_{mi}^{\prime }{\left({\hat{g}}_{mi}^{q}\right)}^{*}s_{i}.$$

The signal-to-interference-plus-noise-ratio (SINR) of the *k*th terminal is

(35) $${\mathrm{SIN}R}_{k}^{q}=\frac{\rho_{ap}{\left(\sum_{m=1}^{M}\sqrt{\eta_{mk}}{\hat{g}}_{mk}^{q}{\left({\hat{g}}_{mk}^{q}\right)}^{*}\right)}^{2}}{\rho_{ap}\sum_{i\neq k}^{K}{\left(\sum_{m=1}^{M}\sqrt{\eta_{mi}}{\hat{g}}_{mk}^{q}{\left({\hat{g}}_{mi}^{q}\right)}^{*}\right)}^{2}+\rho_{ap}\sum_{i=1}^{K}\sum_{m=1}^{M}\eta_{mi}{\hat{g}}_{mi}^{q}{\left({\hat{g}}_{mi}^{q}\right)}^{*}E\left[e_{mk}^{\prime }{\left(e_{mk}^{\prime }\right)}^{*}\right]+1}.$$

The quantization of the estimated channel vector introduces additional quantization error into the channel estimation error. Therefore, more channel error is introduced into the available downlink rate in FDD cell-free massive MIMO after CSI quantization, leading to available downlink rate degradation. At the same time, more quantization bits increase the CSI feedback overhead, which is proportional to the number of quantization bits.

## 5. Results

We assume that 100 APs and 10 terminals are identically and uniformly distributed within a square of size 1×1 km${}^{2}$. The channel model is the 3rd Generation Partnership Project (3GPP) UMi channel model [27]. The noise variance at the receiver is assumed to be $\sigma_{w}^{2}=T_{0}\times \kappa \times B\times NF$, where κ, *B*, and NF are the Boltzmann constant, bandwidth and noise figure, respectively. The pilot overhead $\tau_{p}$ equals *M* for downlink training or *K* for uplink training. Other parameters are summarized in Table 3.

### 5.1. Comparison of Different Schemes

**Figure 4:**
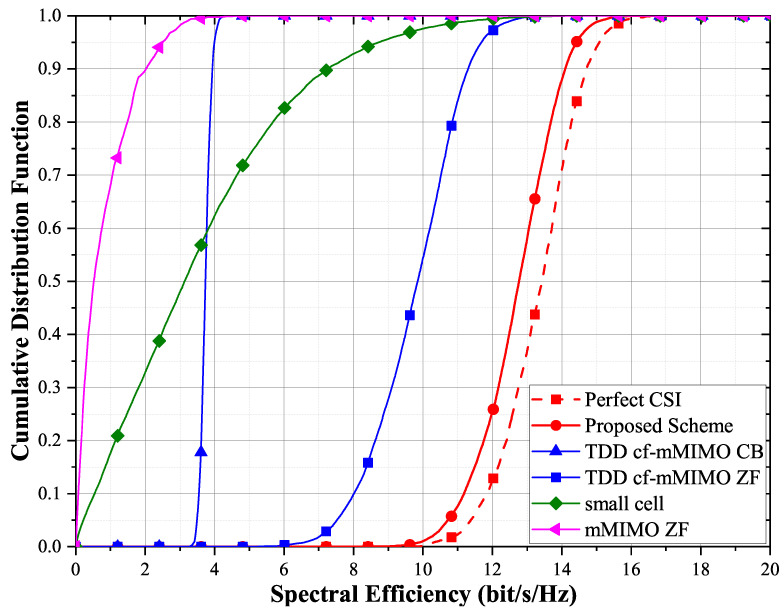
Cumulative distribution function of the achievable per-user rates for different schemes with max–min fair power allocations for M=100 and K=10. $\tau_{p}=100$ for the proposed FDD cell-free massive MIMO (cf-mMIMO), and $\tau_{p}=10$ for the TDD cell-free massive MIMO, small cell and massive MIMO (mMIMO).

Figure 4 compares the spectral efficiency performance of different schemes. The blue curve, TDD cf-mMIMO CB, corresponds to the TDD scheme of reference [8,13], which uses statistical CSI for power control and transmission. Compared with that of the proposed scheme, the performance of the TDD cf-mMIMO CB is significantly worse. The proposed scheme uses instantaneous CSI instead of statistical CSI, and it improves the spectral efficiency by approximately 7.5 bit/s/Hz over the use of statistical CSI, i.e., by approximately three times. Even the ZF scheme using statistical information is not as good as the scheme using instantaneous CSI. The channel is no longer hardened, which has a serious impact on cell-free massive MIMO. Conventional centralized massive MIMO and small cell networks have difficulty in providing uniformly excellent QoS for each terminal under max–min fair power control. This occurs because, in massive MIMO and small cell networks, there are terminals with very poor channel states. The systems incur a considerable cost to guarantee the transmission of the worst terminals. Due to the difference in network structure, the cf-mMIMO system has more advantages than small cell and massive MIMO in providing uniform quality of service. The channel hardening phenomenon is attenuated, and the gain from channel estimation cannot be ignored.

### 5.2. Comparison of Different Numbers of Quantization Bits

**Figure 5:**
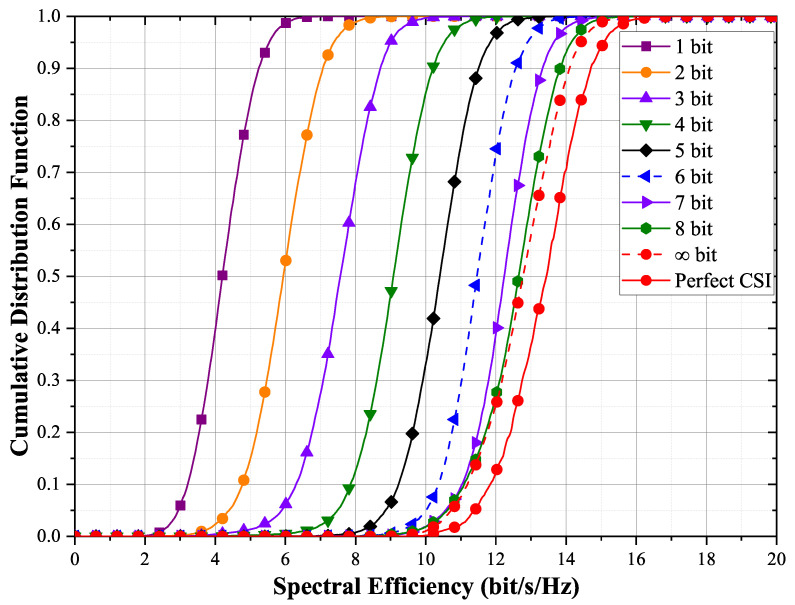
Cumulative distribution function of the achievable per-user rates for different numbers of quantization bits with max–min fair power allocations for M=100 and K=10.

Figure 5 compares the spectral efficiency performance of the estimated channel vectors for different numbers of quantization bits. When the number of quantization bits is less than six, the spectral efficiency is increased by approximately 1–1.7 bit/s/Hz with each additional quantization bit. Compared with full feedback with 32-bit or higher precision coefficients, quantized feedback of channel coefficients loses some performance but greatly reduces the feedback overhead. Even with a 1-bit quantization feedback scheme, the spectral efficiency is close to that of TDD CB scheme, which is in Figure 4. When the number of quantization bits is more than 6 bits, the spectral efficiency improvement is limited. To keep the curves simple, only up to 8 bits are plotted in Figure 5. The performance is close to that of infinite bits when the number of quantization bits is 8 bits. When the number of quantization bits is infinite, the performance is slightly worse than the perfect CSI, and the performance loss is caused by the channel estimation error.

### 5.3. Comparison of Different Numbers of APs and UE

**Figure 6:**
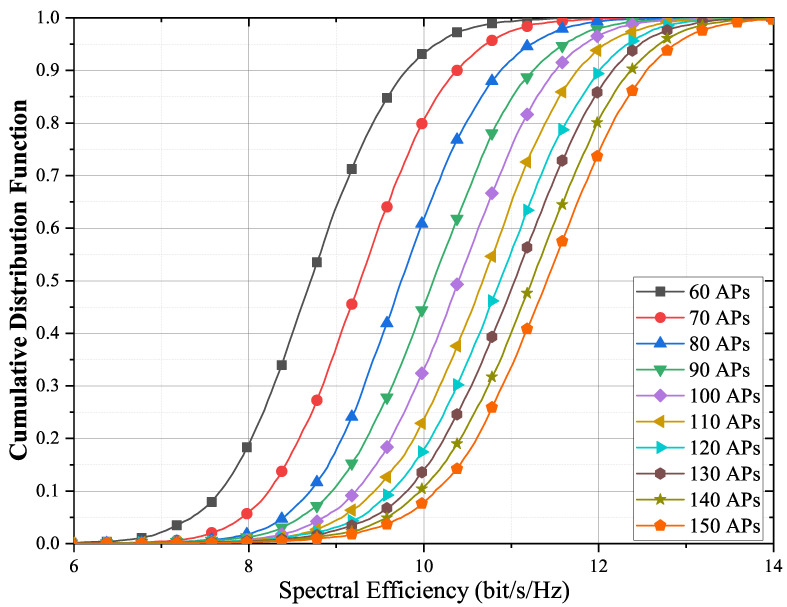
Cumulative distribution function of the achievable per-user rates for different APs with max–min fair power allocations for M=60,⋯,150 and K=10. The number of quantization bits is 5 bits.

Deploying different numbers of antennas in a fixed scenario means that the antenna density varies. It directly affects the average distance from a UE to an antenna. A smaller average distance provides less path loss. Figure 6 compares the impact of the number of APs on the spectral efficiency. The figure shows that the spectral efficiency increases with the number of antennas. When the number of antennas is small, increasing the number of APs gives a significant performance improvement. When the AP density is large, the spectral efficiency gain obtained by further increasing the number of APs is limited.

**Figure 7:**
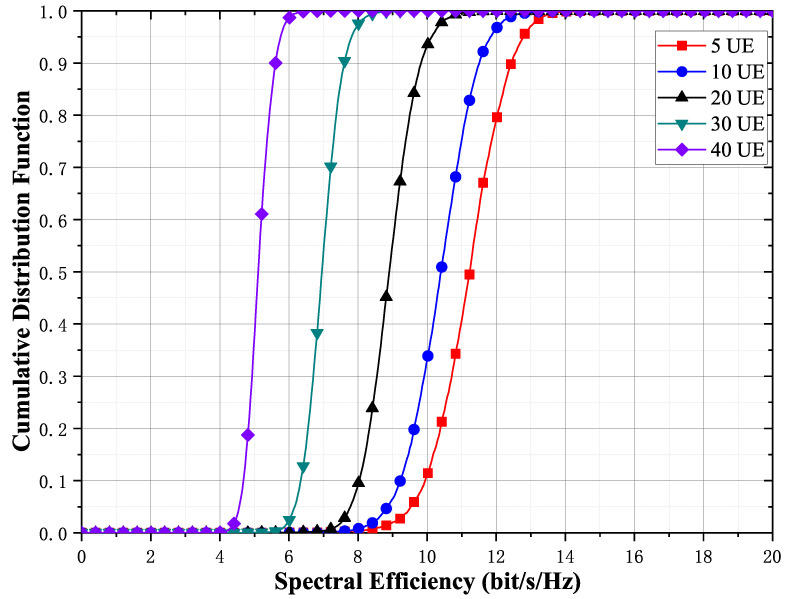
Cumulative distribution function of the achievable per-user rates for different UE with max-min fair power allocations for M=100 and K=5,10,20,30,40. The number of quantization bits is 5 bits.

Figure 7 illustrates the effect of the number of terminals transmitting simultaneously. As the number of terminals increases, the spectral efficiency of each terminal decreases. The 95%-likely spectral efficiency of each terminal is approximately 9.7 bit/s/Hz when the system serves five terminals simultaneously and decreases to approximately 4.5 bit/s/Hz when the number of simultaneously served terminals increases to 40. Although the spectral efficiency of each terminal decreases, the total spectral efficiency of the system is improved. Therefore, increasing the number of simultaneously served terminals can improve the system capacity under the premise of guaranteeing the QoS requirements of terminals.

### 5.4. Comparison of Rural-Macro (RMa), Urban-Macro (UMa), and UMi Scenarios

**Figure 8:**
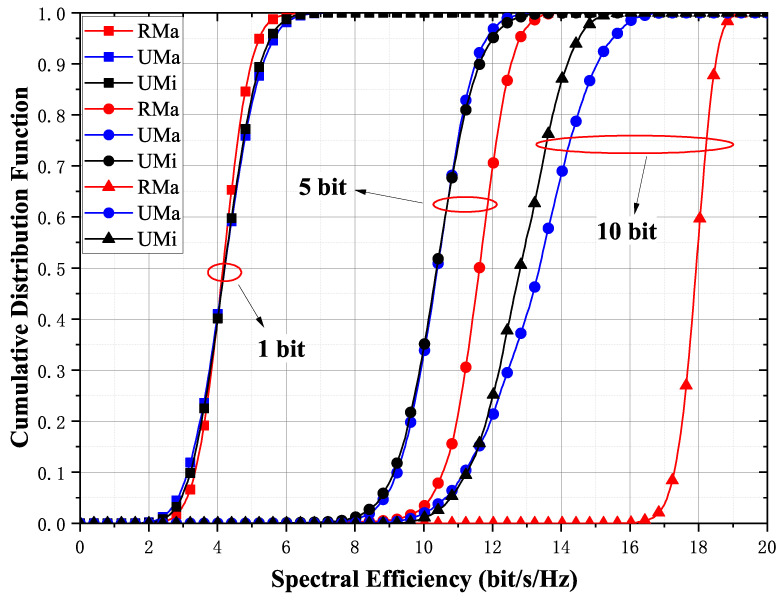
Cumulative distribution function of the achievable per-user rates for different scenarios with max-min fair power allocations for M=100 and K=10. The number of quantization bits is 1, 5, and 10 bits.

In Figure 8, the performance of FDD cell-free massive MIMO is compared in different scenarios. Under the condition of high-precision quantization, the RMa scenario has the highest spectral efficiency due to its smaller path loss and better propagation conditions. This means that, when deployed in practice, UMi and UMa scenarios require more dense AP deployment or design of AP placement to achieve similar performance to RMa scenarios. Compared with UMa and UMi, the 95%-likely spectral efficiency in the RMa scenario is approximately 6 bit/s/Hz higher, while the spectral efficiencies of RMa and UMi are similar. With five quantization bits, the above conclusion still holds, but the performance gap is narrowed. This is because the quantization error becomes larger and therefore results in a loss of performance. With one quantization bit, the spectral efficiency is almost the same in all three scenarios.

## 6. Conclusions

In this paper, we exploit the fact that the channel hardening phenomenon is not evident in cell-free massive MIMO compared with centralized massive MIMO. We propose refraining from the use of the channel hardening phenomenon for cell-free massive MIMO downlink transmission but assume that the CPU and UE obtain CSI in a slowly time-varying channel environment. The available downlink rate is re-derived and combined with max–min power control to solve for the power control coefficient. Furthermore, we propose to quantify CSI to reduce feedback overhead. The simulation results show that when the CSI is known to the terminals, the spectral efficiency increases by a factor of approximately three, compared to transmission with statistical channel information.

## Figures and Tables

**Table 1:** Channel Hardening.

	$M=10^{1}$	$M=10^{2}$	$M=10^{3}$	$M=10^{4}$	$M=10^{5}$
Massive MIMO	0.1005	0.0099	0.001	9.9 $\times \;10^{-5}$	9.98 $\times \;10^{-6}$
Cell-Free	0.6173	0.5152	0.3745	0.1839	0.048

**Table 2:** Favorable Propagation.

	$M=10^{1}$	$M=10^{2}$	$M=10^{3}$	$M=10^{4}$	$M=10^{5}$
Massive MIMO	0.1001	0.0101	9.99 $\times \;10^{-4}$	9.92 $\times \;10^{-5}$	1.009 $\times \;10^{-5}$
Cell-Free	0.1016	0.0107	0.0012	3.385 $\times \;10^{-5}$	6.371 $\times \;10^{-6}$

**Table 3:** Key Simulation Parameters.

Parameter	Value
Transmit Power of APs and Terminals	200 mW
System Bandwidth *B*	20 MHz
Centering Frequency	2.0 GHz
Height of AP and UE Antenna	15 m & 1.65 m
Noise Figure (NF)	9 dB
κ	$1.381\times 10^{-23}$ J/K
$T_{0}$	290 K
